# Clinical Lectures on Diseases of the Ear

**Published:** 1871-07

**Authors:** E. L. Holmes

**Affiliations:** Prof. Ophthalmology and Otology, Rush Med. College, Surgeon to Chicago Charitable Eye and Ear Infirmary


ARTICLE VIII.—*Clinical Lectures on Diseases of the Ear.* By Dr. E. L. HOLMES, Prof. Ophthalmology and Otology, Rush Med. College, Surgeon to Chicago Charitable Eye and Ear Infirmary.

### MECHANISM OF HEARING.

GENTLEMEN—I propose to present for your examination and study a series of cases, which will illustrate the most common pathological changes in the ear. From these cases, I trust you will learn the methods of investigating aural diseases, comprehend the manner in which they may result in the impairment of hearing, and understand the principles on which their treatment is based.

If you will follow me but for a few minutes in the study of the important points in the anatomy and physiology of the ear, and of the principal phenomena of sound, I think you will admit that the obscurity regarding the nature and diagnosis of aural disease has been greatly exaggerated. I think, also, you will be convinced that while the treatment of many of these diseases is still very unsatisfactory, the treatment of many of the diseases is attended with the most gratifying results.

I shall not burden your attention with the minute anatomy of the ear. Leaving this for your private study of our best works, I shall point out the few landmarks which, if carefully fixed in the memory, will render the rest of the field easily mastered.

Disregarding for the present the external ear, glance for a moment through this silver conical tube into the ear. By the aid of a good light falling directly through the tube, or better, by means of light thrown by this concave reflector, you are able to look upon the walls of the membrana tympani, and the walls of the external meatus. This membrane — parchment-like — is stretched across the external meatus, separating this from the middle ear. Were it not for this membrane, the middle ear would be simply a continuation of the external meatus.

The middle ear, or tympanum, is a small cavity in the bone, lined with delicate mucous membrane, which also covers the inside of the membrana tympani. This cavity has at its *upper portion* two openings, one leading forward to the throat, the other (one or more) backward to the mastoid cells.

If you examine a recent preparation of the temporal bone, from which the membrana tympani has been removed, you will observe that you can look upon the inner wall of the middle ear, and can pass the point of a probe through the external meatus upon the little elevation called the promontory. In this inner wall, just posteriorly to the promontory, are two small apertures —the *upper* one, oval—the foramen ovale; the lower one, round —the foramen rotundum. Study and fix in your mind all facts regarding these two minute openings, for they play a most important part in the mechanism of hearing.

In the foramen ovale, is fitted the base of the little bone called the stapes or stirrup, so called from its very great resemblance to the stirrup of a saddle. If the portion corresponding to the part of the stirrup on which the foot rests, were circular, this bone would resemble the "box" or piston of the common house pump, as often constructed. In fact, the stapes performs almost precisely the same function in the ear as the piston in the force pump or syringe. Its oval portion, or base, rests quite loosely in the foramen ovale, a very delicate membrane, passing from the edges of the foramen to the base of the stapes, and serving as packing, prevents the passage of water around it, without interfering with its delicate motions as a piston.

The other two minute bones, the anvil and hammer—incus and malleus—serve the same relative purpose as the shaft of a piston. The incus is attached to the stapes by a very delicate joint, and also to the malleus, which last is united, by means of its long handle, to the inside of the membrana tympani. Pressure on the outside of the membrana tympani is consequently conveyed through these minute bones to the stapes, which is moved to a greater or less extent in the foramen ovale, precisely as pressure on the handle of a syringe is communicated through it to the piston, causing it to move in the cylinder.

We have spoken of *two* openings on the inner side of the cavity of the tympani, as being of the greatest importance in this wonderful mechanism. The foramen ovale has just been described as a minute and short cylinder in which plays the stapes as a piston. Before explaining the function of the other, or foramen rotundum, we must show what lies on the other side of the foramen ovale. On examining a prepared specimen, you find that this opening leads into a very irregular cavity called the labyrinth, the portion of this cavity nearest the stapes being termed the vestibule. There are two minute curved channels, each end of which opens into the vestibule; another minute curved channel passes from the vestibule into one of the other canals, consequently, these three little canals, called the semi-circular canals, have five openings in the vestibule. Connected with the vestibule is a very wonderful little organ called the cochlea. You will comprehend the structure of the cochlea by considering the following illustration. Imagine a small leaden tube, closed at one end—further, imagine this tube divided into two lateral halves by a very thin partition passing from the open end *almost to the closed end* of the tube. You can thus understand how water turned into one lateral half would pass down to the bottom and then ascend in the other lateral half. Now imagine this little tube curled two and a half times upon itself in the form of a spiral, and you have at once an idea of the form of the cochlea. This minute spiral in the ear is so placed that one of its lateral halves opens into the vestibule, the other into the *foramen rotundum*, over which is spread a delicate membrane—the membrana tympani secundaria—separating the middle ear from the cochlear portion of the labyrinth.

The next important point for your consideration is the fact that the labyrinth is filled with a limpid fluid, which lies in contact with the fibers of the auditory nerve. You are well aware that water is practically incompressible. If the fluid in the labyrinth was confined in a cavity wholly surrounded with solid bone, the stapes could have no motion in either direction, on account of the incompressible fluid on one side and the weight of the atmosphere on the other. If you fill a large syringe with water, and place the finger tightly over the orifice from which the stream flows, you will have an illustration of this principle, when you attempt to move the piston in either direction.

In the ear, however, pressure on the membrana tympani, as we have shown, causes the stapes to move inward in the foramen ovale—the fluid is forced through one side of the cochlea, and returns in the same direction through the other half to the foramen rotundum, and causes a slight convexity of its membrane outward. When the stapes is withdrawn, the atmosphere presses on the membrane of the foramen rotundum, and forces the fluid from the cochlea into the vestibule. Hence, one of the chief functions of the foramen rotundum and its membrane is to render vibrations of the stapes possible.

These are, I am well aware, only a very few cardinal points in aural science. But if you will study them carefully, and fix them in your memory, you will, as I said in the beginning, comprehend the more readily the minute anatomy of the ear as you find it in our best text books.

Those of you who are desirous of pursuing the study of aural disease, I must refer to the work of Von Troeltsch, translated by Prof. Roosa, and to the Archives of Ophthalmology and Otology, edited by Profs. Knapp and Moss.

In our next lecture we will consider the mechanism I have described, in its more intimate relation with the phenomena of sound.

